# Neural differentiation potential of human bone marrow-derived mesenchymal stromal cells: misleading marker gene expression

**DOI:** 10.1186/1471-2202-10-16

**Published:** 2009-03-03

**Authors:** Katrin Montzka, Nina Lassonczyk, Beate Tschöke, Sabine Neuss, Tobias Führmann, Rachelle Franzen, Ralf Smeets, Gary A Brook, Michael Wöltje

**Affiliations:** 1 Department of Neurology, RWTH Aachen University, Aachen, Germany; 2 Institute for Neuropathology, RWTH Aachen University, Aachen, Germany; 3 Interdisciplinary Center for Clinical Research (IZKF) 'BIOMAT.', RWTH Aachen University, Aachen, Germany; 4 Department of Biomedical Engineering, RWTH Aachen University, Aachen, Germany; 5 Institute of Pathology, RWTH Aachen University, Aachen, Germany; 6 Research Center for Cellular and Molecular Neurobiology, University of Liège, Liège, Belgium; 7 Clinics for Oral and Maxillofacial Surgery, RWTH Aachen University, Aachen, Germany; 8 Roche Diagnostics GmbH, Penzberg, Germany

## Abstract

**Background:**

In contrast to pluripotent embryonic stem cells, adult stem cells have been considered to be multipotent, being somewhat more restricted in their differentiation capacity and only giving rise to cell types related to their tissue of origin. Several studies, however, have reported that bone marrow-derived mesenchymal stromal cells (MSCs) are capable of transdifferentiating to neural cell types, effectively crossing normal lineage restriction boundaries. Such reports have been based on the detection of neural-related proteins by the differentiated MSCs. In order to assess the potential of human adult MSCs to undergo true differentiation to a neural lineage and to determine the degree of homogeneity between donor samples, we have used RT-PCR and immunocytochemistry to investigate the basal expression of a range of neural related mRNAs and proteins in populations of non-differentiated MSCs obtained from 4 donors.

**Results:**

The expression analysis revealed that several of the commonly used marker genes from other studies like nestin, Enolase2 and microtubule associated protein 1b (MAP1b) are already expressed by undifferentiated human MSCs. Furthermore, mRNA for some of the neural-related transcription factors, e.g. Engrailed-1 and Nurr1 were also strongly expressed. However, several other neural-related mRNAs (e.g. DRD2, enolase2, NFL and MBP) could be identified, but not in all donor samples. Similarly, synaptic vesicle-related mRNA, STX1A could only be detected in 2 of the 4 undifferentiated donor hMSC samples. More significantly, each donor sample revealed a unique expression pattern, demonstrating a significant variation of marker expression.

**Conclusion:**

The present study highlights the existence of an inter-donor variability of expression of neural-related markers in human MSC samples that has not previously been described. This donor-related heterogeneity might influence the reproducibility of transdifferentiation protocols as well as contributing to the ongoing controversy about differentiation capacities of MSCs. Therefore, further studies need to consider the differences between donor samples prior to any treatment as well as the possibility of harvesting donor cells that may be inappropriate for transplantation strategies.

## Background

Mesenchymal stromal cells (MSCs), often termed mesenchymal stem cells, may be isolated from bone marrow, adipose and other tissues. They adhere strongly to tissue culture plastic and are capable of multipotent differentiation that can be demonstrated *in vitro*. MSCs can differentiate into osteoblasts, chondroblasts, adipocytes and myoblasts (reviewed in [1]). Since the survival and migration of human MSCs (hMSCs) grafted into rat brains was demonstrated [2], the possibility that such cells might act as suitable tools for promoting central nervous system (CNS) repair has been raised. This notion was further strengthened by the report that murine MSCs may differentiate into mature astrocytes after implantation into neonatal mouse brains [3]. Donor MSCs have also been reported to give rise to neuronal phenotypes in adult mice brains after transplantation [4,5]. During the last six years, there have been many reports describing *in vitro *neural transdifferentiation of MSCs derived from a range of different species (e.g. human, mouse, rat). All protocols that have been used for such investigations can be divided into three broad categories: i.e. those using (i) chemical compounds [6-10], (ii) growth factors [11-14], or (iii) neurosphere-like cultivation [15-20]. Furthermore, combinations of these different protocols have also been reported to induce neural differentiation [21,22]. Such reports of *in vitro *neural transdifferentiation by MSCs derived from experimental animal- or human sources have been based on the detection of neural-related mRNA as well as proteins by the treated cells. In the present investigation, RT-PCR and immunocytochemistry were used to demonstrate the basal expression of several of the commonly used marker genes or proteins by undifferentiated human MSCs. The data obtained by this study revealed a substantial degree of heterogeneity of the basal expression of neural-related genes that had not previously been described.

## Results

### Characterization of human MSCs

Isolated hMSCs were characterized by three criteria: (i) adherence to tissue culture plastic, (ii) specific surface antigen expression, and (iii) multipotent differentiation as defined by the *Mesenchymal and Tissue Stem Cell Committee of the International Society for Cellular Therapy *[23].

**Figure 1:**
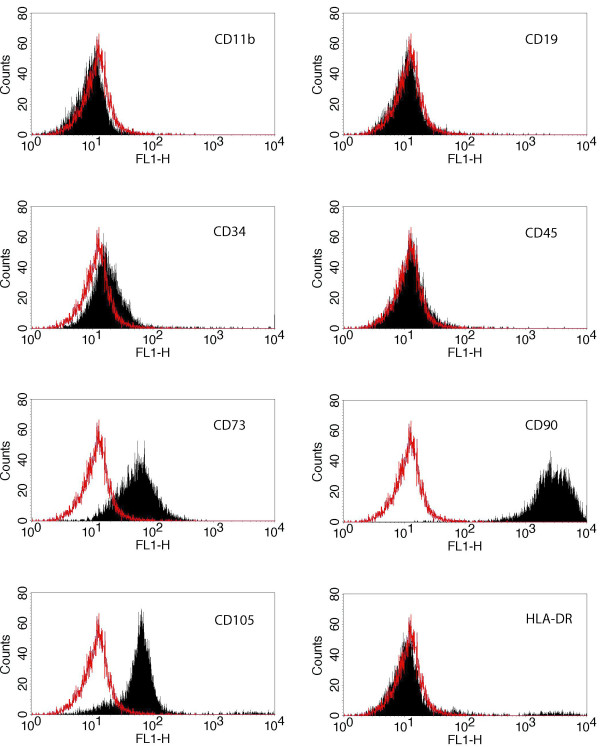
**Surface marker expression of human MSCs**. FACS analysis of the immunophenotypic surface profile for CD11b, CD19, CD34, CD45, CD73, CD90, CD105 and HLA-DR of isolated hMSCs. Red histograms represent the fluorescence from negative-control cells incubated with only secondary antibody; black histograms represent the counts of cells incubated with the relevant primary antibody. The logarithm on the X-axis (FL1-H channel) represents the intensity of the fluorescent signal and the number of cells is given on the Y-axis. HMSCs isolated in this study were positive for the markers CD73, CD90 and CD105, but negative for CD11b, CD19, CD34, CD45 and HLA-DR according to the criteria for MSCs.

**Figure 2:**
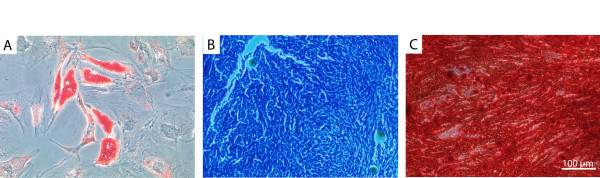
**Differentiation capacity of hMSCs**. The hMSCs were cultivated for 21 days in differentiation media to show multi-potentiality. (A) Adipogenic differentiation resulted in the formation of lipid vacuoles which were stained with Oil Red O; (B) Thin sections of chondrogenic differentiated hMSC-derived cell pellets were stained with Toluidine Blue, demonstrating a highly enriched extracellular matrix; (C) Alician Red staining of hMSCs induced to osteocytes revealed an immense mineral deposition.

Fluorescent activated cell sorting (FACS) analysis demonstrated that the expanded, plastic adherent cells used in the present investigation were positive for the surface markers CD73, CD90 and CD105, but negative for CD11b, CD19, CD34, CD45 and HLA-DR (Figure 1). To demonstrate their multipotent potential, MSCs were differentiated to adipocytes, chondrocytes and osteocytes according to published protocols [24]. Lipid vacuoles in differentiated adipocytes were visualized with Oil Red O (Figure 2A). However, not all cells demonstrated the same degree of staining. Induction of chondrogenic differentiation was performed in cell pellets which developed a proteoglycan-rich extracellular matrix. Thin sections of these pellets were stained with Toluidine Blue (Figure 2B), demonstrating a metachromatic staining that was characteristic of cartilage matrix [25]. Osteogenic differentiation resulted in an immense production of mineral deposits that were stained with Alizarin-Red-S (Figure 2C). Thus, the cells used in this study fulfilled all criteria to be defined as MSCs.

### Neural marker expression by undifferentiated human MSCs

**Figure 3:**
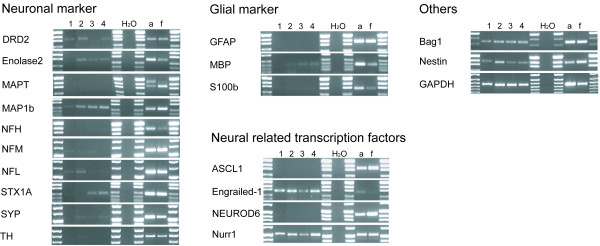
**Neural marker expression of human MSCs**. Neural marker transcripts of four different donors were amplified by RT-PCR. Water served as negative control and commercially obtained adult (a) or fetal (f) brain cDNA was used as positive control. GAPDH was used as standard.

The neural specific RNA transcripts, obtained from hMSCs of four different donors, were categorized into four sub-groups: (i) neuronal markers, (ii) glial markers, (iii) neural related transcription factors and (iv) others (Figure 3, table 1). For every marker, both adult and fetal human brain extracts were used as positive controls.

**Table 1:** Summary of neural-related marker expression

**Marker**	**Donor 1**	**Donor 2**	**Donor 3**	**Donor 4**
DRD2	++	++	-	++
Enolase 2	-	++	+	+
MAPT	-	-	-	-
MAP1b	++	+++	+++	+++
NFH	-	-	-	-
NFM	-	-	-	-
NFL	+	++	-	+
STX1A	-	-	++	++
SYP	-	+	+	+
TH	-	+	-	-

GFAP	-	-	-	-
MBP	-	+	+	+
S100β	-	-	-	-

ASCL1	-	-	-	-
Engrailed-1	+++	+++	++	+++
NEUROD6	-	-	-	-
Nurr1	+++	+++	++	+++

Bag1	+++	+++	+++	+++
Nestin	++	+++	++	++
GAPDH	+++	+++	+++	+++

PCR transcript expression was defined as strong (+++), moderate (++), weak (+) or not detectable (-).

#### Neuronal markers

One of the neuronal markers investigated was dopamine receptor D2 (DRD2), a marker for dopaminergic neurons [26], which was moderately expressed by three donors (donors 1, 2 and 4). Transcripts for enolase2 (or neuron-specific enolase, NSE), normally found in mature neurons and in cells of neuronal origin [27], were also found in human MSCs (donor 2 moderate, donors 3 and 4 weak). Microtubule-associated protein 1b (MAP1b), that is suggested to play an important cytoskeletal role in development and function of the nervous system [28], was strongly detected in donors 2 to 4 as well as moderately in donor 1. Other widely used markers for neurons are the neurofilaments (NF). The expression of NFH (heavy), NFM (medium) and NFL (light) in human MSCs was analyzed in the present study. Low levels of transcripts for NFL could only be detected in donors 1 and 4, but moderate levels were found in donor 2. The syntaxin (STX1A) functions in the fusion of synaptic vesicles with the synaptic membrane of neurons [29]. However, a moderate amount of STX1A was also detected in the undifferentiated MSCs of donor 3 and 4. Another synaptic vesicle-related protein, synaptophysin (SYP) [30], was only slightly detectable in donors 2 to 4. The expression of tyrosine hydroxylase (TH), a neurotransmitter-related enzyme in catecholaminergic neurons, was also only weakly detectable in donor 2. No expression of mRNA for the axonal microtubule-associated protein tau (MAPT, [31]) could be observed (for an overview of the case-by-case expression of the markers genes, see Table 1),

#### Glial markers

Analyzed in this study were glial fibrillary acidic protein (GFAP) [32], myelin basic protein (MBP) [33] and the calcium binding protein, S100β [34]. None of the hMSCs were found to express S100β or GFAP, an intermediate filament protein that is normally expressed by astrocytes and Schwann cells. However, 3 of the hMSC population (donors 2–4) were found to express low levels of mRNA for MBP, a major myelin associated protein.

#### Neural-related transcription factors

Transcripts for achaete-scute complex homolog 1 (ASCL1, also known as Mash1), a transcription factor that plays a role in neuronal commitment and differentiation and in the generation of olfactory and autonomic neurons [35], could not be detected in any of the donor cells. Furthermore, none of the donor cells expressed mRNA for neurogenic differentiation 6 (NEUROD6), a factor suggested to be involved in the development and maintenance of the mammalian nervous system [36]. However, the expression of the homeobox protein Engrailed-1 (important in the formation of interneurons [37] as well as in the regionalization of the developing brain [38]) and Nurr1 (involved in the function of dopaminergic neurons [39]), were detected at moderate (donor 3) and strong levels (donors 1, 2 and 4).

#### Others

The transcripts investigated in this category were Bag1, nestin and also GAPDH (which served as an internal control). Bag1, which has anti-apoptotic functions in a variety of cell types and plays an essential role in the survival of differentiated neurons [40], was highly expressed in all donor samples. The intermediate filament protein nestin, was initially identified as a marker for neural stem cells [41], but has since been found to have a much broader expression in a range of cell types and tissues [42-47]. Transcripts for nestin were also detected in all human donor MSCs samples, however, there were clear differences in the intensity of the signal; MSCs derived from donor 2 being much stronger than that from donors 1, 3 and 4.

The commercially available samples of total RNA that had been generated from adult and fetal human brain samples showed developmentally related changes in the intensity of expression for certain mRNAs, e.g. stronger bands for the neuronal markers Enolase2, NFH, SYP, the glial markers MBP and S100β, and the transcription factor Engrailed-1 were fall elevated in control adult samples. However, weaker bands for MAP1b, nestin and the transcription factors ASCL1 and NEUROD6 were found in adult control samples. No major differences in the intensity of bands between control adult and fetal brain samples could be found for the other transcripts (i.e. DRD2, NFM, NFL, STX1A, TH, GFAP, Nurr1 and Bag1). The differences in mRNA transcripts resemble the expected shifts in the intensity of expression during the development of the nervous system. The transcription factors of the basic helix-loop-helix family ASCL1 and NEUROD6 are expressed in neuronal progenitors [48,49] and the cytoskeletal constituents nestin and MAP1b were expressed at higher levels in the control fetal samples. On the contrary, Enolase2 [27], NFH [50], SYP [30], MBP [33] and S100β [51] are expressed by more mature cells, reflecting their higher level of expression in the control adult samples. Although the transcription factor Engrailed-1 plays a role in the formation of interneurons as well as in the regionalization of the developing brain [38], it is possible that the developmental stage from which the control fetal sample was obtained had already passed the time point for expression. Alternatively, expression of the transcript is only found in a fraction of cells and is diminished in total brain mRNA extraction. The faint Engrailed-1 band detected in the adult samples may have reflected its anti-apoptotic role in mature neurons [52].

### Immunocytochemical detection of neural marker proteins by undifferentiated human MSCs

**Figure 4:**
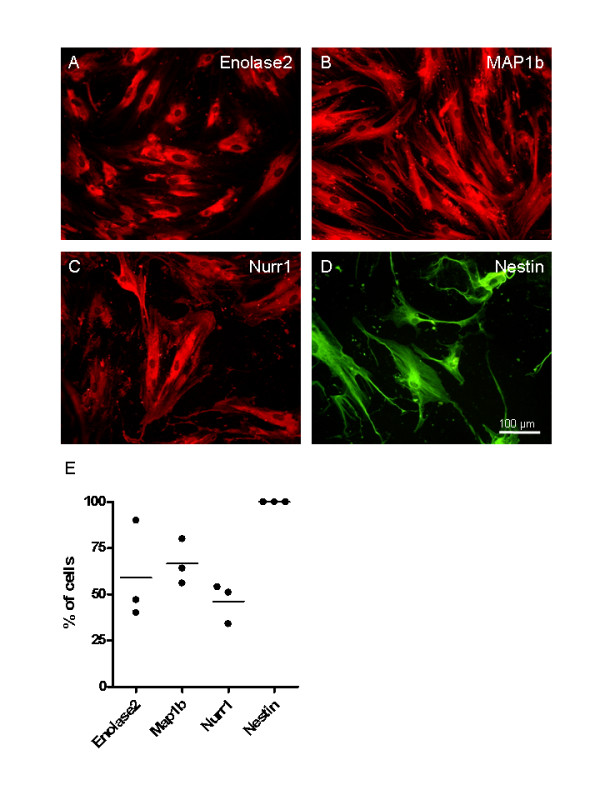
**Expression of neural related proteins**. Immunofluorescence for neural related proteins in undifferentiated hMSCs. (A) Enolase2, (B) MAP1b, (C) Nurr1 and (D) nestin. Staining revealed cytoplasmic distribution of Enolase2 and Nurr1, whereas the staining of MAP1b and nestin was cytoskeletal. Scale bar 100 μm. (E) Quantification of the percentage of stained cells from three different donors revealed following data: Enolase2 expression was found in 59% ± 27.1% of all cells, 66.7% ± 12.2% Map1b positive cells, and 46.3% ± 10.8% of the cells expressed Nurr1. Nestin expression was found to be present in all cells analyzed.

In addition to RT-PCR, a number of non-differentiated donor hMSC samples were chosen for immunocytochemical analysis. Staining of Enolase2 revealed a cytoplasmic distribution (Figure 4A). A cytoskeletal staining was observed with antibodies against MAP1b (Figure 4B) and nestin (Figure 4D). However, staining for Nurr1 was detected at different intensities and revealed a cytoplasmic distribution (Figure 4C). Quantification of immunocytochemical staining from three donors (Figure 4E) revealed that Enolase2 expression was found in 59 ± 27.1% of all cells, Map1b expression was found in 66.7 ± 12.2% of all cells, and Nurr1 expression was found in 46.3 ± 10.8% of the cells expressed. Nestin expression was found to be present in all cells of the three donors analyzed. Thus, this data demonstrates that both protein and mRNAs of a range of neurally-related markers is already expressed by non-differentiated hMSC.

## Discussion

The potential of adult MSCs to transdifferentiate into neural cell types has aroused great interest in research. Such a capacity opens extensive possibilities for autologous therapeutic treatments in a variety of neurological disorders. However, clear and unequivocal data regarding differentiation needs to be generated to provide a solid foundation for further studies. In the present investigation, undifferentiated hMSCs were found to express a number of neural-related genes (albeit at rather low levels for some mRNAs). Significantly, it became clear that there was considerable donor-related heterogeneity in the expression pattern of the hMSC populations, even though the criteria for isolation and identification (as set out by the *Mesenchymal and Tissue Stem Cell Committee of the International Society for Cellular Therapy *[23]) were followed. It is possible that such donor heterogeneity might be responsible for the variable degree of success reported by a number of research groups in their ability to transdifferentiate hMSCs to a neural phenotype.

One of the first descriptions of neural differentiation by hMSCs reported the detection of nestin and GFAP after differentiation, but importantly failed to report the pre-differentiated phenotype of the cells [11]. This same oversight has also occurred in a most recent publication demonstrating the generation of neural cell types from human bone marrow-derived MSCs [53]. Neurospheres derived from human MSCs were shown immunocytochemically to express nestin and Musashi-1, however, the basal expression profile of the MSCs was not reported. Rapid morphological changes of rat MSCs have also been reported to be associated with the detection of enolase2 and NFM (amongst other markers [6]). However, the non-treated, control cells were not analyzed and the rapid morphological changes aroused great scepticism because they did not reflect normal developmental processes [54]. Such changes were later demonstrated to be due to the breakdown of the actin cytoskeleton [55].

Previous attempts to identify the basal expression pattern of undifferentiated human MSCs using serial analysis of gene expression (SAGE) has revealed the presence of mRNA not only characteristic for mesenchymal cells but also endothelial, epithelial and neuronal lineages [56]. Neural related mRNAs found to be amongst the 50 most abundant gene products expressed included high molecular weight neurofilament (NFH), the high affinity nerve growth factor receptor (TrkA) and glial derived nexin 1 alpha. However, in stark contrast, NFH was undetectable in all donor hMSC samples of the present investigation. This observation likely reflects the high degree of donor sample heterogeneity mentioned earlier.

Heterogeneity of gene expression by rat and human MSCs has also reported following RT-PCR of cell populations and clonal cell lines, revealing mesodermal, germinal, endodermal and ectodermal expression patterns [57,58]. Neurally related transcripts expressed by rat MSC included NMDA receptor sub-units, syntaxin, amyloid precursor protein, and both rat and human MSCs were reported to express GFAP and NeuroD mRNA [57,58]. Both of these transcript were absent in all of the samples used in the present investigation. However, Syntaxin 1A was weakly expressed in 2 of the human samples but was negative in the remaining 2 donor samples, once again highlighting the substantial inter-donor variability.

In the present investigation, the expression of nestin mRNA could be found in all donor samples of undifferentiated hMSCs. Nestin was originally identified as an intermediate filament protein expressed by neural stem cells [59]. Recent reports using rat MSCs have described undifferentiated MSCs as being nestin-negative, but that expression increased progressively with increasingly higher passage numbers [15]. The acquisition of nestin expression by the higher passage number MSCs has been suggested to be an important stage in the ability of the cells to form spheres and subsequently undergo neural differentiation [15]. It is possible that such progressive increase of nestin expression may reflect the culture conditions selecting for – and enhancing the proliferation of a sub-population of nestin-positive cells. The presence of such sub-populations of stem cells or progenitors within MSCs may contribute to the high degree of heterogeneity reported by others [56-58,60,61]. Indeed both intrinsic plasticity of MSCs and contamination by stem cells from other sources (neural crest-derived stem cells) have been suggested to be contributory mechanism to the apparent switch to a neural phenotype by MSCs [58,62]. Although recent publications have demonstrated the expression of a number of neural-related genes in non-differentiated population or clones of MSCs, the degree of donor variability has, until now, remained unclear.

Transplantation of undifferentiated MSCs into experimental models of CNS injury has clearly demonstrated improved motor and sensory function [63-65], and in a phase I clinical study, transplantation was proven to be safe [66,67]. Following engraftment, donor MSCs were reported to be associated with a range of neuronal and glial markers suggesting the spontaneous differentiation of the grafted cells *in vivo *[5,68-70]. Since it was shown, that the spontaneous differentiation of grafted cells occurs only in few cells [68], other reasons for inducing a beneficial outcome should be considered. An on-going discussion revolves around the possible improvement in function being an outcome of cell fusion events between donor MSCs with host cells [71,72]. However, cell fusion also occurs at only a very low frequency [72] and it has been described that neural differentiation can occur without this phenomenon [15,19]. An alternative, and possibly more likely, mechanism for improved function after implantation of MSCs may be due to the local release of growth factors [73,74]. It has been shown that undifferentiated MSCs express a range of growth factors including neurotrophin-3 (NT-3), brain-derived neurotrophic factor (BDNF), glial-derived neurotrophic factor (GDNF), nerve growth factor (NGF), vascular endothelial growth factor (VEGF), hepatocyte growth factor (HGF) [75], ciliary neurotrophic factor (CNTF) and basic fibroblast growth factors (bFGF) [74,76,77]. These factors could certainly play a role in several processes, including neurogenesis, neuroprotection, vascularisation and scar inhibition as it was already demonstrated for VEGF [78]. Such a donor heterogeneity was also reported by others for growth factor secretion by hMSCs. Transplantation of cells was found to promote a variable, donor-dependent degree of axon growth and functional recovery [74]. Furthermore, it is possible that donor MSCs may also influence axonal or tissue regeneration in- and around the host lesion site by providing an array of growth-supporting extracellular matrix molecules, including fibronectin, collagens, laminin, hyaluronan and proteoglycans. These molecules are involved in migration, cell survival, cell proliferation and cell differentiation (reviewed in [79]) and a change in their local concentrations may influence endogenous neural stem cells [77].

## Conclusion

The ability of MSCs, in particular human MSCs, to generate neural-related cell types for future transplantation-based intervention strategies has become a topic of considerable controversy. A number of possible mechanisms have been suggested to explain such cellular behaviour, including true transdifferentiation, the presence of multiply-primed stem cells capable of differentiating into a number of lineages, contamination by neural crest-derived stem cells and tissue culture artefacts. The present investigation supports the contention that MSCs selected on the basis of plastic adherence, multipotency and FACS analysis are highly heterogeneous populations of cells. All donor samples investigated in the present demonstrated expression patterns that were different from each other. Such inter-donor variability regarding the expression of neural-related genes has not been previously described, but supports the heterogeneity of hMSCs reported by others. It is possible that such inter-individual variability may affect the ability of donated cells to respond to particular tissue culture conditions. It should therefore be considered that not all donated MSCs may be appropriate for future autograft cell transplantation strategies.

## Methods

### Sampling of human MSCs

Bone marrow was aspirated from patients during hip joint replacement following informed consent, according to local ethical board approval of the University Hospital, Aachen.

### Isolation and cultivation of human MSCs

Bone marrow aspirates were diluted 1:5 in mesenchymal stem cell growth medium (MSCGM, Lonza, Vervieres, Belgium) and immediately seeded into polystyrene plastic 75 cm^2 ^tissue culture flasks at 37°C in 5% humidified CO_2_. After seven days, non-adherent cells were removed by media replacement and adherent cells were expanded in MSCGM. Media exchange was performed every 3–4 days until cells reached 80% confluence. For passaging, the cells were detached with trypsin/EDTA solution (Lonza, Vervieres, Belgium) and re-seeded with a density of 4000 cells/cm^2^.

### Characterization of isolated human MSCs

For characterization of human MSCs, three criteria were used: i) adherence to tissue culture plastic, ii) specific surface antigen expression, and iii) multipotent differentiation potential [23]. The morphology of plastic adherent cells was monitored using an inverse microscope (DM IL Invers, Leica, Wetzlar, Germany). To detect specific surface antigens, cells were detached and fixed with 4% paraformaldehyde for 20 min. After washing with 0.1 M phosphate-buffered saline (PBS), cells were incubated in blocking solution (20% FBS in PBS, Biowest, Nuaille, France) for 20 min. After washing with PBS, the cell pellets (250,000 per antibody) were resuspended in 100 μl PBS, 2 μl primary antibody was added and incubated for 30 min. Monoclonal primary antibodies recognizing surface markers CD11b (Invitrogen, Carlsbad, USA), CD19, CD34, CD45, CD73, CD90 (Becton Dickinson, San Jose, USA), CD105 (Invitrogen) and HLA-DR (Abcam, Cambridge, UK) were used. After three washing steps with PBS, 2 μl of the secondary antibody (Alexa-488 conjugated goat anti-mouse, Invitrogen) was incubated in 100 μl PBS for 30 min in the dark. After two final washing steps, the cells were re-suspended in 400 μl PBS and analyzed using a FACSCalibur and FACSCalibur software (Becton Dickinson).

Multipotency was monitored by *in vitro *differentiation of MSCs to adipogenic, chondrogenic and osteogenic lineages according to Pittenger et al. [24]. To induce adipogenesis, cells were cultivated in adipogenic induction medium (DMEM (Lonza, Vervieres, Belgium) with 10% FCS, 0.5 μM dexamethasone, 0.5 μM indomethacin, and 0.5 mM isobutyl-methyl-xanthine (all Sigma, Steinheim, Germany)). Medium was changed every 3 days for 3 weeks. To visualise lipid droplets, Oil Red O staining (Sigma) was used as a histologic stain.

Osteogenic differentiation was induced by cultivation of MSCs in osteogenic induction medium (DMEM with 10% FCS, 20 nM sodium β-glycerophosphate, 1 nM dexamethasone, and 50 μg/ml 1-ascorbic acid 2-phosphate, all purchased form Sigma). Medium was changed every 3 days for 3 weeks. Osteogenic differentiation was visualised by Alizarin-Red-S staining (Sigma) of matrix mineralization associated with osteoblasts (according to the method of [80]).

For chondrogenic differentiation, 2.5 × 10^5 ^cells were centrifuged to obtain cell pellets. These pellets were induced with serum free induction media (DMEM) containing 100 nM dexamethasone, 0.17 mM 1-ascorbic acid 2-phosphate, 100 μg/ml sodium pyruvate, 40 μg/ml proline (all Sigma), and 1% ITS-Plus (Becton Dickinson). TGF-β3 (CellSystems, Sankt Katharinen, Germany) was added in a concentration of 10 ng/ml at each medium exchange. After 21 days, pellets were fixed with PFA and paraffin embedded. Thin sections were stained with Toluidine blue (Sigma) to show metachromatic staining which is characteristic of cartilage [25,80].

### Gene expression analysis

RNA was isolated using the RNeasy Mini Kit (Qiagen, Hilden, Germany) according the manufacturers protocol. The integrity of isolated RNA was evaluated using the 2100 bioanalyzer (Agilent Technologies, Palo Alto, USA). For PCR analysis 2 μg RNA was reverse transcribed using Omniscript Reverse Transcriptase (Qiagen, Hilden, Germany) according the manufacturers protocol using oligo-d(T) primers and random hexamers. Commercially available samples of human adult and fetal brain total RNA were used as positive controls (Ambion, Austin, USA and Clontech-Takara Bio Europe, Saint-Germain-en-Laye, France). For amplification of target genes, 0.2 units Taq polymerase (Amersham Biosciences, New York, USA) were used with a standard PCR program (denaturation: 10 sec at 96°C; primer hybridisation: 2 min at various target specifc temperatures, see table 2; elongation: 2 min at 72°C). PCR products were analyzed by electrophoresis on a 1.7% agarose gel and visualized with 0.5 μg/ml ethidium bromide. PCR conditions were established using adult and fetal brain cDNA and best results from at least two independent experiments were chosen for expression analysis of hMSCs. Expression of each marker was monitored by testing cDNA of all four donors using the same master mix, PCR condition and gel electrophoresis.

**Table 2:** Primer sequences and PCR conditions

**Primer**	**Forward (5'- 3')**	**Reverse (5'- 3')**	**Hybridization temperature (°C)**	**Cycles**	**Amplicon size (bp)**
**ASCL1**	aag caa gtc aag cga cag cg	agtcgt tgg agt agt tgg gg	62	35	352
**Bag1**	tca ccc aca gca at gaga ag	cag aaa acc ctg ctg gat tc	66	30	344
**DRD2**	tca tcg ctg tca tcg tct tcg	gat gga gat cat gac ggt gac	65	40	344
**Engrailed-1**	agc cac agg cat caa gaa cg	cac ctg tcc gag tct ttc tc	65	40	303
**Enolase2**	ggc aaa ggt gtc ctg aa gg c	gtg ccg gcc ttc aac gtg at	67	30	284
**GAPDH**	tga agg tcg gag tca acg gat ttg gt	cat gtg ggc cat gag gtc cac cac	62	30	983
**GFAP**	gtg gta ccg ctc caa gtt tgc ag	aat ggt gat ccg gtt ctc ctc	59	40	373
**MAP1B**	act gca gga cca gga act ac	cag tgt cac ctg cat gtt gc	67	25	255
**MAPT**	agc tct ggt gaa cct cca aaa tc	cat cca tca taa acc agg agg tg	58	40	361, 454
**MBP**	cct ggc cac agc aag tac	ggg agc cgt agt gag cag t	61	35	251
**Nestin**	gcg ttg gaa cag agg ttg gag	gca cag gtg tct caa ggg tag	65	30	385
**NEUROD6**	ctg aga atc ggc aag aga cc	ctg cac agt aat gca tgc cg	62	35	433
**NFH**	tga aca cag acg cta tgc gct cag	cac ctt tat gtg agt gga cac aga g	58	35	398
**NFL**	acc aac gag aag caa gcg ctc	cat cag cgc tat gca gga cac	59	35	590
**NFM**	aaa gac atc gag gag gcg tc	cgc tgc gta cag aaa act cc	61	35	592
**Nurr1**	aag gct tct tta agc gca cag	cga tta gca tac agg tcc aac	55	25	518
**S100β**	gga gac aag cac aag ctg aag	agc tac aac acg gct gga aag	63	30	322
**STX1A**	atc gca gag aac gtg gag gag	agc gtg gag tgc tgt gtc ttc	67	30	230
**SYP**	ggt gct gca atg ggt ctt cgc	aag ccg aac acc acc gag gtg	59	35	537
**TH**	atc cac cat cta gag acc cg	tcc ccg ttc tgc tta cac ag	63	40	824

### Immunocytochemistry

The hMSCs were seeded at a relatively low density to allow the clear identification of individual cells for quantification. After removal of the media and washing in 0.1 M phosphate buffered saline (PBS), cells were fixed with 4% paraformaldehyde in PBS for 30 min. Afterwards, the samples were washed three times with PBS and non-specific binding sites blocked by a 1 hour incubation in PBS containing 3% normal goat serum (Sigma), 1% bovine serum albumin (BSA, fraction 5, Serva, Heidelberg, Germany) and 1% Triton X-100 (Sigma). The primary antibodies anti-enolase2 (monoclonal, Dako M0873; 1:500), anti-MAP1b (monoclonal, Sigma M4528; 1:500), anti-Nurr1 (monoclonal, Abnova H00004929-M07; 1:200) and anti-nestin (polyclonal, Chemicon AB5922; 1:200) were diluted in PBS containing 1% BSA and incubated at room temperature overnight. The secondary antibodies, goat-anti-mouse Alexa 594 (1:500, Invitrogen) and goat-anti-rabbit Alexa 488 (1:500, Invitrogen) were also diluted PBS containing 1% BSA and incubated for 2.5 hours at room temperature. Finally, nuclei were stained with diamidinophenylindole (DAPI, 1:1000, Roche), cover-slipped with Fluoroprep (bioMerieux, Marcy l'Etoile, France) and observed using a Leica DM RX microscope (Leica, Wetzlar, Germany) with 20× objective. Omission of primary antibodies served as negative control and resulted in no detectable staining. Immunocytochemistry of cells obtained from three different donors was performed on 2 separate occasions. The proportion of cells immunoreactive for a particular antigen was quantified by counting 100 cells per donor sample. To achieve this, 10 randomly chosen, non-overlapping microscopic fields (each 577 μm × 465 μm) distributed evenly across the whole sample were photographed to provide representative images of the stained and non-stained cells. The 10 fields were believed to be representative of whole donor samples because the immunocytochemistry revealed that there was no heterogeneity of the distributions of the stained and non-stained cells – i.e. there were no clusters of stained cells or non-stained cells.

## Authors' contributions

KM, NL and BT contributed to the design of the experiments, to isolation of bone marrow stromal cells and cell culture; KM contributed the adipogenic and osteogenic differentiation and drafted the manuscript; KM and BT contributed the flow cytometric analysis; SN contributed the chondrogenic differentiation of human MSCs. NL contributed the RT-PCR; TF and GB contributed the immunofluorescence; RF contributed the MAP1b staining; RS contributed to taking and isolation of human bone marrow stem cells. MW designed the study and was involved in the writing of the manuscript. All authors read, corrected and approved the final manuscript.
